# Fungal Pericarditis—A Systematic Review of 101 Cases

**DOI:** 10.3390/microorganisms13040707

**Published:** 2025-03-21

**Authors:** Predrag Jancic, Stefan Milutinovic, Marshall Ward, Milan Radovanovic, Nikola Jovanovic, Marina Antic, Nikola Nikolajevic, Marija Petrovic, Dorde Jevtic, Adam Adam, Igor Dumic

**Affiliations:** 1 Mayo Clinic Health System, 1221 Whipple Street, Eau Claire, WI 54703, USA; prjancic@gmail.com (P.J.); radovanovic.milan@mayo.edu (M.R.); antic.marina@mayo.edu (M.A.); nikicke.nn@gmail.com (N.N.); 2 Internal Medicine Residency Program, Florida State University, Tallahassee, FL 32301, USA; smilutinovic@fsu.edu; 3 Dartmouth Hitchcock, Hanover, NH 03766, USA; marshall.ward@hitchcock.org; 4 Department of Pediatrics, University of Nis, 18000 Nis, Serbia; nikola92.jovanovic92@gmail.com; 5 Mount Sinai Hospital, New York, NY 10029, USA; marija.petrovic@mountsinai.org; 6 Internal Medicine Residency Program, Elmhurst Hospital, New York, NY 11373, USA; jevticd@nychhc.org; 7 Cook County Hospital, Chicago, IL 60612, USA; adam.adam@cookcountyhealth.org

**Keywords:** pericarditis, fungal, *Candida*, *Aspergillus*, *Mucor*, *Cryptococcus*, fungemia, immunosuppression

## Abstract

Fungal pericarditis is a rare disease but its incidence has risen in parallel with the global increase in invasive fungal infections. This systematic review analyzes data from previously reported cases of fungal pericarditis to provide an improved understanding of the etiology, clinical presentation, management, and outcomes of this rare disease. We reviewed Medline and Scopus databases from 1 January 1990 to 29 January 2024 for case reports that documented the isolation of a fungal pathogen from pericardial fluid or tissue. Of the 2330 articles screened, 101 cases met the inclusion criteria. Patients with fungal pericarditis and the involvement of at least one other organ—usually the lungs, brain, or kidney—had worse outcomes than patients with isolated pericardial disease. Immunosuppression was reported in 50% of cases and was associated with worse outcomes in adults. Patients who presented with chest pain, received adequate empiric antifungal therapy, and underwent pericardiocentesis and pericardiectomy had improved survival. The most common isolated pathogens were *Candida* spp., followed by *Aspergillus* spp. and *Mucor* spp., with the latter two linked to worse outcomes. Only 35% of patients received empiric antifungal medications before the causative pathogen was identified, and mortality was associated with a delay in appropriate therapy. Immunosuppression, disseminated disease, and presence of shock/multiorgan failure were additional risk factors associated with death. Fungal pericarditis carries a mortality rate of up to 50%, with nearly half of patients being immunocompromised. Clinicians frequently do not consider fungal pericarditis in the differential diagnoses, which leads to delays in treatment and poorer outcomes. Further prospective multicenter studies are urgently needed to better understand the epidemiology, improve diagnostic testing and management, and decrease unacceptably high mortality in patients with fungal pericarditis.

## 1. Introduction

Pericarditis, defined as inflammation of the pericardial sac, is the most common disease of the pericardium [1,2,3]. It can be characterized as non-infectious or infectious, with viruses being the most common infectious etiology [1]. Bacterial and fungal pathogens can also cause pericarditis, with *Mycobacterium tuberculosis* isolated more frequently in under-resourced countries [4]. Fungal pericarditis occurs when yeasts or molds invade the pericardial space and is indicative of invasive fungal infection. The pericardium can be involved directly through injury or surgery, or by continuous spread from the surrounding structures or through hematogenous spread. The global incidence of invasive fungal infections has risen significantly over the past decades, largely driven by immunosuppression as a major contributing factor [5,6,7,8,9]. The most common invasive infections identified are those caused by *Candida* spp., *Aspergillus* spp., *Cryptococcus* spp., and *Mucor* [10]. Furthermore, atypical fungal species, mostly environmental fungi that are historically not associated with human infection, have been on the rise in recent years, significantly contributing to the changing epidemiology of fungal infections [11].

Despite advancements in awareness and non-invasive diagnostic tests, diagnosing invasive fungal infections remains challenging. Timely and accurate diagnosis is crucial, as delays are strongly associated with poor outcomes [12,13]. Treatment follows multisociety guidelines and relies on antifungal drugs; however, rising resistance threatens efficacy and cure rates [11,14,15,16,17,18]. Moreover, the optimal duration of treatment for fungal pericarditis remains undefined due to a lack of clinical trials. Invasive fungal infections are more common in immunocompromised patients who are at a particular risk; however, infections have also been reported in immunocompetent patients [19,20]. The precise incidence of fungal pericarditis remains unknown. This study aims to systematically analyze data from previously published cases of fungal pericarditis to provide a more comprehensive understanding of the clinical presentation, management, and outcomes of this rare disease.

## 2. Materials and Methods

We performed a systematic review of the literature according to the Preferred Reporting Items for Systematic Reviews and Meta-Analyses (PRISMA) guidelines using Medline (National Library of Medicine, Bethesda, MD, USA) via the PubMed search engine, and the SCOPUS database, from 1 January 1990 to 29 January 2024. All included cases had documented isolation of a fungal pathogen from pericardial fluid, biopsy, or autopsy.

The justification behind the timeframe after the year of 1990 is twofold: firstly, case reports dating before 1990 were hard to find in their full-text form, and secondly, the timeframe was chosen to reflect the therapeutic and diagnostic modalities used today, making any statistically significant findings more likely to be relevant today.

**Figure 1:**
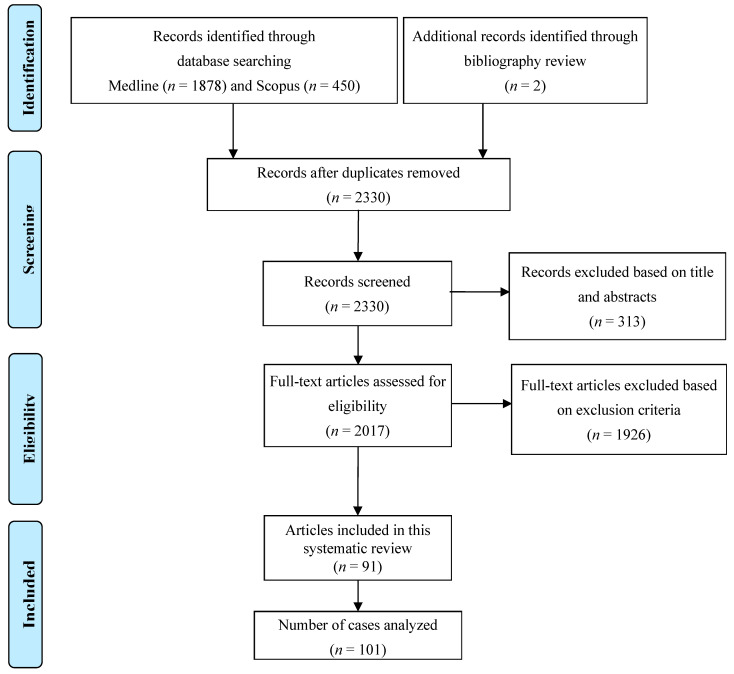
PRISMA flow diagram of the study selection process.

This study is registered with the Research Registry, and the unique identifying number is registry1947. A total of 1878 original articles from Medline and 450 articles from Scopus were identified that mention MeSH terms “pericarditis OR myopericarditis OR pericardium OR pancarditis” AND “fungal OR fungus OR fungi OR candida OR aspergillus OR aspergillosis OR histoplasma OR histoplasmosis OR blastomyces OR blastomycosis OR coccidioidomycosis OR paracoccidioidomycosis OR basidiobolomycosis OR cryptococcus OR cryptococcal OR mucormycosis OR sporothrix”. We excluded cases where the diagnosis was uncertain either because pericardiocentesis, pericardial biopsy, or autopsy was not performed, or pericardial fluid was sterile. We also excluded duplicate articles, articles in languages other than English, abstracts without comprehensive case descriptions, and narrative reviews. Ultimately, our review included 101 cases that fulfilled the criteria. Figure 1 shows the detailed process of article selection and the final cases included in the analysis.

Four authors (M.A, M.R, S.M., and P.J.) independently and blindly identified and selected titles, abstracts, and full texts in the database search. Discrepancies in the selected articles were resolved by the senior author (I.D.). Subsequently, the reference list of selected articles was searched to identify any additional articles for inclusion in accordance with previously established selection criteria. The following data were extracted: patients’ demographic data, co-morbid conditions, presence of immunosuppression, presenting symptoms, treatment strategies, complications, outcomes, and laboratory and imaging findings including electrocardiography, echocardiography, and computerized tomography scans. Immunosuppression was reported as one of three categories: absent, presence of cancer and/or cancer treatment, and history of organ transplantation and other (autoimmune disease, HIV infection, congenital disease, etc.). Comorbid conditions were categorized into nine categories: absent, cancer diagnosis, history of organ transplantation, liver cirrhosis, history of non-thoracic surgery, presence of hypertension and/or coronary artery disease, autoimmune and/or rheumatic conditions, genetic conditions, and presence of renal failure and/or chronic kidney disease and other.

### Statistical Analysis

Data were analyzed by descriptive and analytical statistics using SPSS statistical software (IBM SPSS statistics, Version 21.0, SPSS Inc., Chicago, IL, USA), and are expressed as the mean ± standard deviation for normally distributed data, or as frequency and percentages for categorical data. The Student’s test and chi-square test were used to compare data between the patients regarding survival. The logistic regression analysis (univariate) was used to assess predictors of survival. A *p*-value <0.05 was considered statistically significant.

We considered the initial diagnosis and treatment as appropriate if the authors suspected fungal infection on admission and if the appropriate empiric therapy was started within 48 h from admission.

## 3. Results

### 3.1. Demographics, Comorbidities, and Risk Factors for Invasive Infection

**Figure 2:**
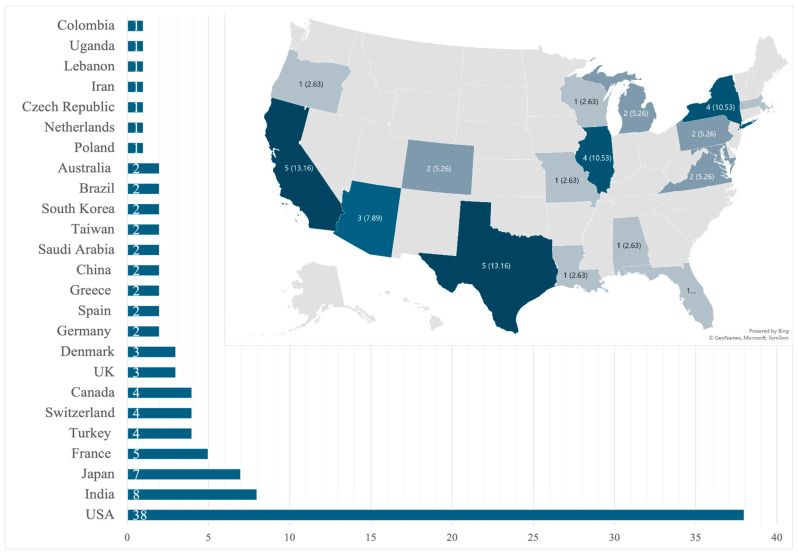
Global and state-by-state distribution of cases within the United States.

Our review identified 101 unique patients that fulfilled the inclusion criteria from 91 case reports describing a single patient and 5 case series that described two patients each [12,13,20,21,22,23,24,25,26,27,28,29,30,31,32,33,34,35,36,37,38,39,40,41,42,43,44,45,46,47,48,49,50,51,52,53,54,55,56,57,58,59,60,61,62,63,64,65,66,67,68,69,70,71,72,73,74,75,76,77,78,79,80,81,82,83,84,85,86,87,88,89,90,91,92,93,94,95,96,97,98,99,100,101,102,103,104,105,106,107,108,109,110,111,112]. A total of 12 cases were pediatric (0 to 18 years) and 89 were adults (18 and older) with an adult mean age of 50.3 ± 16.7 years, with that for pediatrics being 5.8 ± 5.5 years. Most patients were male (*n* = 68, 67.3%) and no statistical differences were seen in sex-related survival (*p* = 0.337 for adults; *p* = 0.224 for the pediatric population). By region, North America reported the most cases (*n* = 42, 41.6%), of which the USA had 38 cases (Figure 2).

A history of immunosuppression was reported in 51 (50.5%) patients (Table 1). In adults, 44 (49.4%) cases reported immunosuppression, which was more frequently observed in deceased patients compared to survivors (27 vs. 17; *p* = 0.02). Among the pediatric population, there was no statistically significant difference in survival associated with immunosuppression. Adult patients who underwent pericardiocentesis, underwent pericardiectomy, and received the appropriate initial diagnosis and treatment had better outcomes (*p* < 0.05), while disseminated disease, presence of complications, and isolation of *Aspergillus* spp. and *Mucor* spp. were associated with worse outcomes (*p* < 0.05). Patients’ demographic data are presented in Table 1, while variables related to the outcome are presented in Table 2.

Risk factors for invasive fungal infection include a history of hematological malignancies, bone marrow or solid organ transplantations, prolonged intensive care unit (ICU) stay, and prolonged neutropenia, among others [113,114]. Half of the patients with fungal pericarditis were immunosuppressed, with the most common causes being a history of bone marrow and solid organ transplantation (*n* = 16, 31.4%) and cancer (*n* = 14, 27.4%). The majority of cancer patients had hematological malignancy (*n* = 11, 57.9%), while the other three cases had either gastric and esophageal cancer and *Candida* spp. that spread contiguously to the pericardium [34,61,94]. All but three adult cases had active cancer treated with chemotherapy at the time of diagnosis, while of the 12 pediatric cases, 3 (25%) had immunosuppression due to cancer treatment, while 2 (16.7%) had a primary immunodeficiency due to chronic granulomatous disease.

In this review, 5 cases of rare fungi (*Trichosporon japonicum*, *Trichosporon inkin*, *Trichoderma longibrachiatum*, *Scedosporium prolificans*, *Curvularia*) were seen exclusively in patients with lung or heart transplantation (4 and 1 cases, respectively) [23,32,44,53,62].

### 3.2. Presenting Signs and Symptoms

Chest pain was reported in 36 (44.4%) adult and 2 (16.7%) pediatric cases, fever in 45 (50.7%) adult and 11 (91.7%) pediatric cases, and dyspnea or tachypnea in 45 (50.7%) adult and 8 (66.7%) pediatric cases. Other commonly reported symptoms included epigastric discomfort and pain, nausea, and vomiting.

### 3.3. Etiology, Other Organ Involvement, and Diagnosis

**Figure 3:**
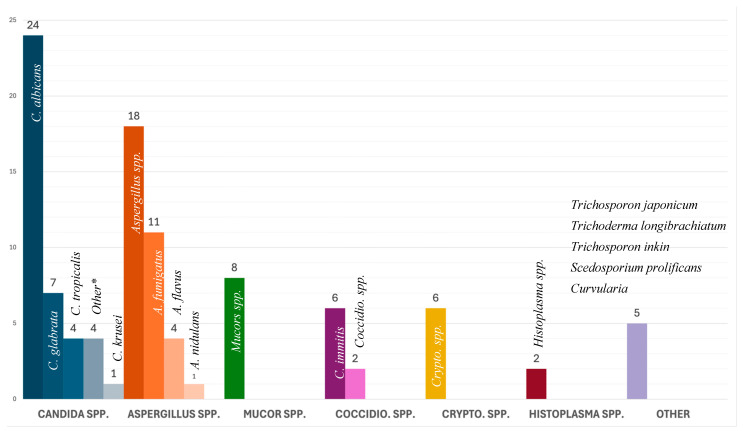
Causative agent by group and number. * Other Candida: *C. parapsilosis* (*n* = 2), *C. novrvegensis* (*n* = 1), and *C. guilliermondii* (*n* = 1).

In adults, *Candida* spp. (*n* = 34, 38.2%) were the most common fungal isolate with *C. albicans* as the dominant species (*n* = 20, 22.5%). In pediatric cases, *Aspergillus* spp. (*n* = 7, 58.3%) were the most common fungal isolate, followed by *Candida* spp. (*n* = 4, 33.3%). Two cases reported a co-infection, one with *C. albicans* and *C. galbrata*, and another with *C. albicans* and *C. tropicalis*. Infection with *Aspergillus* spp. and *Mucor* spp. was statistically associated with lower survival rates (*p* = 0.008). All isolated fungi are presented in Figure 3.

Isolated fungal pericarditis was found in 31 (30.7%) cases, while 70 (69.3%) cases had at least another organ involved: most commonly the lungs (*n* = 43, 42.6%), brain (*n* = 16, 15.8%), and kidney (*n* = 11, 10.9%). Involvement of the other organs was reported in 61 of the 89 adult cases (68.5%) and was associated with death (recovered vs. deceased: 27 vs. 34; *p* = 0.002). Time to diagnosis was inconsistently reported in adults and never in pediatric cases; however, in the cases where patients were promptly diagnosed with fungal pericarditis (14.6%), there was an associated lower mortality (84.6% vs. 15.4%; *p* = 0.011).

A concomitant presence of endocarditis was reported in 4 (4.5%) adult and 2 (16.7%) pediatric cases, and myocarditis in 12 (13.5%) adults and 1 (8.3%) pediatric case. Combined inflammation of the endocardium, myocardium, and pericardium was diagnosed in 10 (11.2%) adults and 1 (8.3%) pediatric case. Blood cultures were reported in 34 (33.7%) cases, of which 8 yielded a positive result (5 *Candida*, 2 *Cryptococcus neoformans*, and 1 *Aspergillus case*). There was no observed association of the presence of fungemia and patient survival rates (alive vs. deceased: 0.0% vs. 3.4%, *p* = 0.249 for adult cases; 16.7% vs. 8.3%, *p* = 1.00 for pediatric cases).

### 3.4. Treatment and Interventions

The most commonly prescribed antifungal was amphotericin B (*n* = 29, 28.7%), followed by fluconazole (*n* = 21, 20.8%), voriconazole (*n* = 17, 16.8%), capsofungin (*n* = 7, 6.9%), itraconazole (*n* = 5, 4.9%), flucytosine (*n* = 4, 4.0%), and other (posaconazole, anidulafungin, micafungin, terbinafine, 5-fluorocytosine, isavuconazole). Of the 68 cases that reported administering antifungal therapy, 44 (64.7%) reported monotherapy and 24 (35.3%) reported either combined therapy or switched between different medications. Procedural interventions included pericardiocentesis in 38 adult (42.7%) and 7 pediatric cases (58.3%), and pericardiectomy in 25 adult (28.0%) and 4 pediatric (33.3%) cases. In adults, both pericardiocentesis (27 vs. 11, *p* = 0.002) and pericardiectomy (22 vs. 3, *p* < 0.001, Table 3) were associated with improved survival. In pediatric cases, this statistical difference was not observed (*p* > 0.05). Recurrence of the pericardial effusion after initial evacuation was observed in 15 cases (14.9%).

### 3.5. Risk Factors for Death, and Complications

In the univariate analysis, pericardiocentesis, pericardiectomy, symptoms of chest pain, and appropriate initial diagnosis were independent predictors of survival in patients with fungal pericarditis, while in multivariate analysis, pericardiocentesis was the only variable that was associated with survival (*p* < 0.05).

Statistically significant risk factors for death included immunosuppression, disseminated disease, development of complication, and infection caused by *Aspergillus* spp. and *Mucor* spp.

Complications were reported in 56 cases (55.5%), with the most common being fungal embolization (*n* = 14 13.9%), followed by shock (*n* = 13, 12.9%), heart failure (*n* = 11, 10.9%), pneumonia/respiratory failure (*n* = 9, 8.9%), multiorgan failure (*n* = 6, 5.9%), and stroke (*n* = 4, 4.0%). Shock, multiorgan failure, stroke from infective emboli, and respiratory failure were significantly associated with death (*p* < 0.001), with 84% of patients dying (Table 2).

## 4. Discussion

### 4.1. Epidemiology, Demographics, and Risk Factors for Fungal Infection

The rising incidence of fungal infections is closely linked to the growing population of older and immunosuppressed individuals [5,6,7,8,9]. The two most common fungi associated with pericarditis in this review are common commensal microorganisms (*Candida* spp.), or ubiquitous in nature (*Aspergillus* spp.) [118,119,120,121,122]. These infections have become a public health emergency due to a limited choice of antifungal medication, an increase in antimicrobial resistance, a lack of vaccines, and a growing at-risk population (older and immunosuppressed) [14,15,16,17,18].

Large-scale epidemiological data on fungal pericarditis are lacking in the available literature [2,123]. The disease is rare enough that even retrospective single-center studies have not been conducted, and the current understanding of this disease is based on case reports and case series. The literature from Sub-Saharan Africa and Asia reports on the more prevalent tuberculosis pericarditis [124], with fungal cases seldom reported. One review from 2009 showed that of 660 cases of purulent pericarditis, only 1.4% were caused by fungi [36]. Our review shows a predominance of fungal pericarditis case reports from high-income countries (Figure 2), which may reflect the lack of diagnostic testing in under-resourced nations, leading to underreporting and publication bias [125,126,127,128,129,130].

Invasive fungal infections have a high mortality rate with approximately 1.5 million deaths annually, over 80% of which are caused by three main fungal species, *Candida* spp., *Aspergillus* spp., and *Cryptococcus* spp. [113,131], findings that were consistent in this review on fungal pericarditis as well. The highest mortality in patients with fungal pericarditis is due to *Mucor* (88%), followed by *Aspergillus* spp. (71%), *Cryptococcus* spp. (33%), and *Candida* spp. (24%) [131].

Interestingly, unlike other invasive fungal infections that primarily affect immunocompromised individuals, 50% of the patients with fungal pericarditis in this review did not present any of the conditions typically associated with immunosuppression. This finding is significant, as it may lead clinicians to overlook fungal pericarditis in immunocompetent populations.

Other risk factors uniquely associated with invasive candidiasis were skin and mucosal barrier defects, gastrointestinal perforations or surgery, and the presence of indwelling intravenous catheters [114]. Surgery, specifically cardiac, gastric and esophageal, is a known risk factor for the formation of gastro-pericardial or esophago-pericardial fistulas which promote contiguous fungal spread and increase the risk for development of fungal pericarditis [132]. Additionally, manipulations of the pericardial and mediastinal space through thoracotomies and pericardiotomies have previously been linked to the fungal invasion [68] of *Candida* spp. into the pericardial space [12,68]. In our review, almost a third (*n* = 12) of pericarditis cases secondary to *Candida* spp. reported some form of thoracic surgery or intervention, while nine cases (23.7%) had a gastrointestinal pathology such as gastric cancer or esophageal fistulas.

### 4.2. Diagnostic and Treatment Challenges

Diagnosing pericardial fungal infections is often challenging. Pericardial fluid is a sterile space, and the gold standard for diagnosis is documenting positive fungal culture from the pericardial fluid or fungal visualization on a biopsy or autopsy specimen. Imaging, such as Computed Tomography (CT) and Magnetic Resonance Imaging (MRI), can aid in diagnosis, but alone, it is not sufficient in establishing the diagnosis [133,134,135]. Blood cultures for fungemia have low sensitivity as noted in a recent review of *Aspergillus* spp. liver abscesses [136,137]. In this review, we found that fungal blood cultures were conducted in 36 (35.6%) cases, out of which 8 (22.2%) were positive. In comparison, patients with bacterial pericarditis, such as Methicillin-resistant *Staphylococcus aureus* (MRSA) pericarditis, had bacteremia in 64% of cases [116]. While the sensitivity of bacterial cultures for diagnosing bacterial pericarditis is higher than in cases of fungal pericarditis, it is still considered low, and molecular techniques of pathogen identifications are preferrable due to the higher diagnostic yield [138,139]. The exact reason for a low blood culture sensitivity for fungal pathogens is unclear but might be due to the test itself, and depends on a variety of factors, including the amount of blood taken and whether the patient was on antifungal medication at the time of culture [140,141,142]. Nonetheless, blood cultures in purulent pericarditis are still considered a key diagnostic tool, belonging to the first class of the level of evidence in the European Society of Cardiology (ESC) guidelines for management of pericardial diseases [123]. In patients with fungal pericarditis, the absence of fungemia does not rule out disease and negative blood cultures should be interpreted cautiously.

Interestingly, the rates of disseminated disease (fungal infection confirmed at least two organs) were similar in patients who had fungemia and those without (25.0% vs. 21.4%). These numbers further show that blood cultures may not be as reliable when assessing the dissemination of fungal infection as they are for bacterial infections.

Serum markers such as galactomannan (GM) and beta-D-glucan (BDG) have different sensitivities and specificities in diagnosing fungal infections, depending on the patient population. Galactomannan has the highest sensitivity in neutropenic patients and those who received allogeneic stem cell transplantation [143]. In non-neutropenic patients, GM tends to have a much lower sensitivity; this also includes solid-organ-transplant recipients [143,144]. Beta-D-glucan follows a similar pattern, with the test exhibiting poor sensitivity and specificity and being used only as an accessory diagnostic tool in some cases [32,144,145,146,147].

In this review, only 4 (4.0%) reported galactomannan and 1 (1.0%) reported beta-D-glucan positivity. All five tests were seen in pericarditis due to *Aspergillus* spp. Many cases omitted serologic testing in their case description altogether. Given these low numbers, we have not been able to make any conclusion on sensitivities of these markers.

### 4.3. Clinical Presentation

Compared to other causes of pericarditis, symptoms seem to vary as displayed in Table 3. Fever was the most common presenting symptom in 56 (55.4%) cases. It was frequent in pediatric patients (*n* = 11, 91.7%) compared to adults (*n* = 45, 50.7%). In comparison, a literature review of influenza myopericarditis that analyzed a similar number of cases showed that 94.7% of patients presented with fever [115]. Chest pain in our review was reported more commonly in the adult (*n* = 36, 44.4%) compared to the pediatric (*n* = 2, 16.7%) population [115]. In the mentioned influenza myopericarditis review [115], 77.3% of the cases had influenza myocarditis accompanying pericarditis, while in our review, only 25 (24.7%) cases reported myocardial involvement, possibly explaining the lack of other common signs of myopericarditis such as tachycardia, hypotension, and shock. As illustrated in Table 3, patients with fungal pericarditis tend to have fever and chest pain less often compared to viral, bacterial, and mycobacterial causes, which might contribute to delayed diagnosis and poorer outcomes.

The lack of pathognomonic signs and symptoms specific to fungal infection might lead to an increase in morbidity and mortality due to a delay in appropriate diagnosis and timely treatment [148,149]. This review showed that the most common causative agent, *Candida* spp., presented with symptoms such as chest pain, fever, and dyspnea in 47.4%, 55.3%, and 55.3% of the cases, respectively, leading to the conclusion that half of the cases had unspecific symptoms to guide diagnosis. Such a delay in timely diagnosis is evidenced in this review, by only 16 (15.84%) of cases correctly assessing the infection as fungal within 48 h of presentation. Additionally, only 36 (35.6%) cases included antifungal medication in their initial empirical treatment, before isolating the causative agent. These numbers suggest that clinicians had a low suspicion of fungal infection. This report also showed that patients had a significantly better survival if there was a high suspicion of fungal infection on presentation and if they were treated timely (*p* = 0.011), highlighting the importance of raised awareness for fungal infections, particularly in immunocompromised patients.

### 4.4. Outcome

Fungal pericarditis has a high rate of mortality of 50% (*n* = 49), which is likely related to the fact that 50.5% of patients who develop fungal pericarditis are immunosuppressed [92,150]. The low index of suspicion, delay in diagnosis, and lack of empiric treatment are other factors that influence high mortality in this group. Despite possessing fewer complications (such as shock and tamponade), patients with fungal pericarditis have markedly higher mortality rates compared to other infectious etiologies (Table 3).

## 5. Conclusions

Fungal pericarditis is a rare but life-threatening condition associated with a 50% mortality rate. Notably, in this review, half of the affected individuals were immunocompetent, underscoring the need for heightened clinical suspicion beyond immunosuppressed populations. Delayed diagnosis and suboptimal empirical treatment contribute to poor outcomes, as fungal pericarditis is often overlooked in differential diagnosis. Patients with fungal pericarditis tend to have a fever and chest pain less often compared to viral, bacterial, and mycobacterial causes, which might contribute to delayed diagnosis and poorer outcomes. Patients with disseminated disease, caused by *Aspergillus* spp. and *Mucor*, and those with complications such as disseminated disease, multiorgan failure, and shock experience significantly worse outcomes. Despite the high mortality, fungemia was rarely reported and the majority of cases did not report galactomannan and beta-D-glucan testing. Given these challenges, there is an urgent need for multicenter studies to enhance our understanding of the epidemiology, clinical presentation, and management of this condition. Future research should prioritize incorporating prospective case registries, recruit larger multicenter datasets, and develop more sensitive and specific biomarkers to facilitate timely diagnosis by clinicians and improve outcomes.

### Limitations of This Study

This systematic review has several limitations. First, we included only English language publications indexed in the MEDLINE and SCOPUS databases. Although stringent criteria were established to exclude low-quality case reports, this approach may have inadvertently omitted high-quality cases that did not meet our pre-selection criteria. Second, due to ongoing updates in microbial nomenclature, multiple names for the same microorganism may appear in the literature. Since this review pooled articles from a wide timeframe, some fungal names may be outdated and relevant articles might have been unintentionally missed. Third, as a systematic review of case reports and case series, our findings are inherently susceptible to publication bias and variability in data reporting. Fourth, the rarity of fungal pericarditis resulted in a small dataset, which limits the broad generalizability of our findings. Lastly, the limited number of pediatric case reports reduces the statistical power of the results and complicates the direct comparison of adult and pediatric cases.

## Figures and Tables

**Table 1:** Demographic characteristics and comorbidities.

Age	*n*	Age Range (Years)	Mean Age (Years)
Pediatric	12 (11.9%)	0.04–17	5.8 ± 5.5
Adult	89 (88.1%)	18–79	50.3 ± 16.7
Total	101 (100.0%)	0.04–79	44.7 ± 21.3
Sex	*n*	%	
Male	68	67.3	
Female	30	29.7	
Not reported	3	3.0	
Immunosuppression	*n*	%	
Not reported	50	49.5	
Reported	51	50.5	
Transplant	16	31.4	
Cancer treatment	14	27.4	
Other *	21	41.2	

* Other: human immunodeficiency virus (HIV) infection, autoimmune, congenital disease, prematurity, vasculitis, transfusion-related disease, and myeloproliferative disorder.

**Table 2:** Patient characteristics associated with outcome in univariate analysis. Symptoms of chest pain, appropriate initial diagnosis, pericardiocentesis, and pericardiectomy (presented in italics in the table) were associated with improved survival. Immunosuppression, infection caused by *Aspergillus* spp. and *Mucor* spp., and development of complications (bolded in the table) were associated with death.

Variable	Alive (*n*)	Dead (*n*)	*p*-Value
Male gender	30 (49.2%)	31 (50.8%)	0.268
**Immunosuppression**	**17 (38.6%)**	**27 (61.4%)**	**0.022**
Chest trauma	17 (65.4%)	9 (34.6%)	0.111
Comorbidities	32 (50.0%)	32 (50.0%)	0.628
Fever	22 (50.0%)	22 (50.0%)	0.086
Dyspnea	25 (56.8%)	19 (43.2%)	0.225
*Chest pain*	*22 (61.1%)*	*14 (38.9%)*	*0.043*
Abnormal ECG	3 (75.0%)	1 (25.0%)	0.675
Pericardial effusion	32 (69.6%)	14 (30.4%)	0.342
Recurrence of pericardial effusion	10 (76.9%)	3 (23.1%)	0.054
Tamponade	13 (61.9%)	8 (38.1%)	0.978
Fungemia	1 (20.0%)	4 (80%)	0.249
Pancarditis	0 (0.0%)	10 (100.0%)	0.078
*Pericardiocentesis*	*27 (71.0)*	*11 (29.0%)*	*0.002*
**Aspergillus and Mucor isolation**	**9 (26.5%)**	**25 (73.5%)**	**0.008**
*Appropriate initial diagnosis*	11 (84.6%)	2 (15.4%)	0.011
*Pericardiectomy*	22 (88.0%)	3 (12.0%)	<0.001
**Disseminated disease**	**27 (44.3%)**	**34 (55.7%)**	**0.024**
**Complications (shock/MOF, RF, stroke/emboli)**	**5 (15.6%)**	**27 (84.4%)**	**<0.001**

Abbreviations: MOF—multiorgan failure; RF—respiratory failure; ECG—electrocardiogram.

**Table 3:** Comparing selected variables based on pericarditis etiology.

Pericarditis Etiology	Age	Comorbidities; Immunosuppression	Fever	Chest Pain	Pericardial Effusion	Tamponade	Shock	Death
Viral [115]	32.3 ± 18.8	42.7%; NR	94.7%	48%	82.7%	50.7%	72%	14.7%
Bacterial [116]	38.5 ± 23.0	86.7%; 69.2%	61.5%	38.5%	94.9%	83.8%	59%	20.5%
Tuberculous [117]	NR	NR; NR	70%	76%	40–60%	NR	NR	NR
Fungal	44.7 ± 21.3	81.2%; 50.5%	55.4%	37.6%	51.5%	21.8%	12.9%	48.5%

Abbreviations: NR—not reported.

## Data Availability

All data are publically available and all case reports used in this research have been appropriately cited and can be accessed through appropriate databases.
